# A 46-year-old female presenting with worsening headache, nuchal rigidity and a skin rash in varicella zoster virus meningitis: a case report

**DOI:** 10.4076/1757-1626-2-6299

**Published:** 2009-09-01

**Authors:** Anurag Kushawaha, Neville Mobarakai, Jill Tolia

**Affiliations:** Department of Medicine, Division of Infectious Diseases, Island University Hospital475 Seaview Ave., Staten Island, NY 10305USA

## Abstract

Varicella zoster virus causes two distinct clinical diseases. Varicella is the primary infection and results from exposure of a person susceptible to the virus. The virus remains latent in cranial nerve ganglia, dorsal root ganglia, and autonomic ganglia along the entire neuraxis. Years later, in association with a decline in cell-mediated immunity in the elderly and immuno-compromised, varicella zoster virus reactivates and can cause a wide range of neurologic disease, including herpes zoster (‘shingles’), postherpetic neuralgia, vasculopathy, myelopathy, retinal necrosis, cerebellitis, and zoster sine herpete. Herpes zoster is associated with numerous neurologic complications and varied presentations. Patients who have a dermatomal distribution of varicella zoster virus and who have headaches should be considered to have VZV meningitis. Virologic confirmation requires testing the cerebrospinal fluid for varicella zoster virus deoxyribonucleic acid via polymerase chain reaction. The application of polymerase chain reaction to the cerebrospinal fluid can be used to detect varicella zoster virus deoxyribonucleic acid and, therefore, infections of the central nervous system. We present a case report of a 46-year-old female who initially presented with worsening headache, nuchal rigidity, fever, and a skin rash, who was subsequently found to have varicella zoster meningitis.

## Case presentation

A 46-year-old Caucasian female of Irish-American descent presented to the emergency department (ED) with a one-day history of worsening headache, nuchal rigidity, and fever. She also complained of photophobia, nausea, one episode of vomiting, and backache.

The patient’s past medical history was significant for nephrolithiasis. The patient denied any medications at home or allergies. She denied any family history of heart disease, cancer, or hypertension. The patient denied smoking, alcohol use, or intravenous drug use. Immunizations were up to date.

She had been vacationing in Massachusetts for two months prior to admission. She spent a significant time outdoors and swimming. She denied insect bites.

Since the past ten days, she had experienced significant pain under her left breast, 7/10 pain scale, with a vesicular eruption occurring for three days.

Review of systems was notable for headache, neck stiffness, the vesicular skin rash and fever.

Vital signs in the ED were noted to be: blood pressure = 150/83 mmHg, temperature = 101°F, respirations = 18 breaths per minute, and pulse = 87 beats per minute. Patient was awake, alert, and oriented to person, place, and time. Physical exam revealed a unilateral vesicular rash extending from T8-T9 dermatomal pattern to under the left breast. Nuchal rigidity was noted. Heart, lung, and abdominal exams were within normal limits.

Upon admission, the patient had a WBC count of 5.2 × 10^3^ cells/ µL with normal chemistries.

Computed tomography scan of the head showed no evidence of acute infarct, hemorrhage, or mass effect. A lumbar puncture was done and the patient was started empirically on intravenous ceftriaxone and vancomycin, oral acyclovir, and dexamethosone. The cerebrospinal fluid (CSF) was also sent to the department of health for Lyme IgM / IgG antibodies, West Nile Virus IgM / IgG, Herpes Simplex Virus 1, 2 DNA PCR and Varicella Zoster Virus (VZV) DNA.

The CSF was noted to be clear / colorless and contained 10 RBCs/mm^3^, 127 WBCs/mm^3^ (100% lymphocytes), glucose 49 mg/dL (serum level, 130), protein 125 mg/dL. The CSF gram stain showed no organisms, with a negative India ink stain and negative CSF bacterial cultures. Acyclovir was continued and antibiotics were stopped.

The initial blood cultures were negative for growth. Serologies for ehrlichia and babesia antibodies were negative. Cerebrospinal fluid testing for Borrelia, WNV, and HSV 1 and 2 were also negative.

By the third day of hospitalization, the patient reported feeling markedly better with reduced headache, neck stiffness and was afebrile. The vesicular rash still persisted and now was associated with pain.

On the fifth day of hospitalization, the patient continued to improve and prednisone was discontinued. The next day, the patient was discharged on a course of oral acyclovir.

Several days after the patient was discharged, the department of health reported that the patient’s CSF was positive for VZV DNA. The patient was contacted at home. She reported to be in good health and denied further meningeal signs and fever.

## Discussion

Varicella zoster virus (VZV) causes two distinct clinical diseases. Varicella, is the primary infection and results from exposure of a person susceptible to the virus. The virus remains latent in cranial nerve ganglia, dorsal root ganglia, and autonomic ganglia along the entire neuraxis. Years later, in association with a decline in cell-mediated immunity in the elderly and immunocompromised, VZV reactivates and can cause a wide range of neurologic disease, including herpes zoster, postherpetic neuralgia, vasculopathy, myelopathy, retinal necrosis, cerebellitis, and zoster sine herpete [1]. Although varicella is mainly a childhood disease, VZV is associated with serious complications, such as CNS involvement, pneumonia, secondary bacterial infections, and death [2]. Older age, immunocompromised state, and possibly pregnancy are risk factors associated with higher severity of varicella [3]. The danger of dying from varicella is highest in infants and in elderly individuals. Although varicella is more severe in immunocompromised persons, most cases of severe morbidity and mortality are seen in healthy people [4].

Most patients are over 60 or immunocompromised [5]. Bone marrow transplant recipients and HIV-positive patients are at particular risk. Not surprisingly, zoster in otherwise young, healthy individuals may be the first manifestation of HIV infection [6].

Extracutaneous sites of involvement include the central nervous system (CNS), as manifested by meningo-encephalitis or encephalitis. The clinical presentation is similar to other viral infections of the brain. Several studies conducted in patients with herpes zoster have demonstrated that subclincal meningeal irritation, indicated by a reactive CSF pleocytosis can occur in many cases [7]. Herpes zoster can be associated with subtle signs of aseptic meningitis - mild CSF mononuclear pleocytosis with slight increase in protein levels - in up to 50% of patients. The application of PCR to the CSF can be used to detect VZV DNA and, therefore, infections of the CNS. The opportunity to study the characteristics of different viral infections of the CNS has been aided by viral PCR of CSF. While CSF protein levels greater than 1000 mg/dL are considered unusual in viral meningitis, in a recent retrospective study, patients with VZV CNS infection had significantly higher CSF protein levels (median > 974 mg/dL) than did patients with enteroviral CNS infection [8] A rare manifestation of CNS involvement by herpes zoster is granulomatous cerebral angiitis, which usually follows zoster ophthalmicus [9].

Acute inflammatory demyelinating polyneuropathy (AIDP, or Guillain-Barre syndrome) is also a rare neurologic complication of varicella and herpes zoster [10]. Patients can have symptoms that are similar to other AIDP that follow other infections.

Myelitis is a common complication following herpes zoster and is sometimes referred to as “postinfectious myelitis”. Spinal cord involvement can occur in almost 50% of patients with spinal (cervical, thoracic, and lumbosacral) herpes zoster [11]. Patients can present with paraparesis, impaired sensation with a level compatible with the segment of VZV reactivation, and sphincter dysfunction. Nevertheless, in the majority of patients, the involvement of the spinal cord is subtle and asymptomatic, and complete recovery is usually achieved.

Another form of VZV myelitis has come to be associated with AIDS patients and is often insidious, progressive, and sometimes fatal. Myeltis in HIV-infected patients involves the spread of VZV from the dorsal root ganglia centrally into the spinal cord parenchyma. CSF exam reveals findings typical of viral encephalitis. Magnetic resonance imaging (MRI) can show longitudinal, serpiginous enhancing lesions. Diagnosis is confirmed with the presence of VZV DNA in the CSF via PCR. Several reports describe zoster myelitis in HIV-infected patients in the absence of any rash and have documented VZV DNA within spinal cord specimens at autopsy [12]

Large vessel granulomatous arteritis is usually a disorder of immunocompetent individuals and can follow herpes zoster or varicella [13]. It is mainly a disorder of the elderly where a brain infarction develops weeks to months following ipsilateral trigeminal zoster and is associated with a mortality of up to 25% [14]. Cerebral aneurysms and hemorrhage can also develop from viral invasion of the vessels. The CSF can show mild lymphocytic pleocytosis, increased protein, sometimes oligoclonal bands, and PCR is positive for VZV nucleic acid. In cases of VZV vasculopathy, the CSF does not always contain PCR-amplifiable VZV DNA, but does contain anti-VZV IgG [15]. Because of the high mortality rate, testing via PCR for both VZV DNA and anti-VZV IgG should be done. Angiography reveals focal narrowing or occlusion of the blood vessel which on autopsy displays arterial inflammation with multinucleated giant cells, Cowdry type A inclusion bodies, and VZV nucleic acid [16].

Small vessel multifocal vasculopathy usually occurs in immunocompromised patients. It usually occurs without any skin lesions and consists of subacute multifocal neurological deficits along with headache, fever, mental status changes, and seizures [17]. A history of herpes zoster may precede the symptomatology by several weeks to months. Cerebrospinal fluid analysis is similar to that of large vessel granulomatous arteritis. The MRI typically demonstrates multifocal infarcts and autopsy reveals mixed necrotic and demyelinative lesions with small vessel vasculopathy.

Focal motor weakness is another neurologic complication that may follow herpes zoster. Motor impairment may follow herpes zoster anywhere from one day to several months and usually involves the same segment that was affected by the rash. Arm weakness and diaphragmatic paresis has been associated with herpes zoster in the cervical dermatomes [18]. Lumbosacral herpes zoster has been associated with leg weakness. Neurogenic bladder and loss of anal sphincter control can follow sacral herpes zoster [19]. Peripheral facial weakness has been reported with herpes zoster oticus. The prognosis of focal motor weakness is generally fair with more than half of patients regaining full motor power [20].

In our patient, the argument can be made that the acyclovir could have been given intravenously. However, a common bedside challenge is to determine whether a case of meningitis has a bacterial or viral etiology, as this will effect antimicrobial selection, which in turn, has its risks and benefits. Seriously ill patients with suspected viral meningitis should probably receive intravenous antiviral therapy. Oral acyclovir can be tried on less severely ill patients, although data on efficacy are lacking. Oral acyclovir was chosen in our patient as she was hemodynamically stable and did not have focal neurological deficits.

Also, in our patient, the CSF glucose/serum glucose ratio was low. Glucose in the CSF normally approximates 60% of the serum glucose level. Low CSF glucose is often seen with bacterial meningitis and is also one of the hallmarks of meningeal carcinomatosis as well as CNS sarcoidosis. The CSF glucose level is typically normal during viral infections. However, low CSF glucose levels can occasionally be observed with mumps, enteroviruses, lymphocytic choriomeningitis, as well as herpes simplex and varicella zoster viruses [21]. This aspect of our case makes it atypical of most cases of VZV meningitis.

In conclusion, herpes zoster is associated with a wide range of neurologic complications and presentations. Patients who have a dermatomal distribution of VZV and who have headaches should be considered to have VZV meningitis. Virologic confirmation requires testing the CSF for VZV DNA via PCR.

## Abbreviations

AIDS: Acquired Immunodeficiency Syndrome
CSF: cerebrospinal fluid
DNA: deoxyribonuceleic acid
HIV: Human Immunodeficiency Virus
HSV: Herpes Simplex Virus
PCR: Polymerase Chain Reaction
RBC/mm^3^: red blood cells per cubic millimeter
WBC/mm^3^: white blood cells per cubic millimeter
WNV: West Nile Virus
